# Transcriptome-Based Identification of ABC Transporters in the Western Tarnished Plant Bug *Lygus hesperus*

**DOI:** 10.1371/journal.pone.0113046

**Published:** 2014-11-17

**Authors:** J. Joe Hull, Kendrick Chaney, Scott M. Geib, Jeffrey A. Fabrick, Colin S. Brent, Douglas Walsh, Laura Corley Lavine

**Affiliations:** 1 USDA-ARS, Arid Land Agricultural Research Center, Maricopa, Arizona, United States of America; 2 USDA-ARS, Daniel K. Inouye Pacific Basin Agricultural Research Center, Hilo, Hawaii, United States of America; 3 Dept. of Entomology, Washington State University, Pullman, Washington, United States of America; Institute of Vegetables and Flowers, Chinese Academy of Agricultural Science, China

## Abstract

ATP-binding cassette (ABC) transporters are a large superfamily of proteins that mediate diverse physiological functions by coupling ATP hydrolysis with substrate transport across lipid membranes. In insects, these proteins play roles in metabolism, development, eye pigmentation, and xenobiotic clearance. While ABC transporters have been extensively studied in vertebrates, less is known concerning this superfamily in insects, particularly hemipteran pests. We used RNA-Seq transcriptome sequencing to identify 65 putative ABC transporter sequences (including 36 full-length sequences) from the eight ABC subfamilies in the western tarnished plant bug (*Lygus hesperus*), a polyphagous agricultural pest. Phylogenetic analyses revealed clear orthologous relationships with ABC transporters linked to insecticide/xenobiotic clearance and indicated lineage specific expansion of the *L. hesperus* ABCG and ABCH subfamilies. The transcriptional profile of 13 LhABCs representative of the ABCA, ABCB, ABCC, ABCG, and ABCH subfamilies was examined across *L. hesperus* development and within sex-specific adult tissues. All of the transcripts were amplified from both reproductively immature and mature adults and all but LhABCA8 were expressed to some degree in eggs. Expression of LhABCA8 was spatially localized to the testis and temporally timed with male reproductive development, suggesting a potential role in sexual maturation and/or spermatozoa protection. Elevated expression of LhABCC5 in Malpighian tubules suggests a possible role in xenobiotic clearance. Our results provide the first transcriptome-wide analysis of ABC transporters in an agriculturally important hemipteran pest and, because ABC transporters are known to be important mediators of insecticidal resistance, will provide the basis for future biochemical and toxicological studies on the role of this protein family in insecticide resistance in *Lygus* species.

## Introduction

ATP-binding cassette (ABC) proteins are an extensive family of transmembrane proteins that are ubiquitous to all organisms. The defining characteristic for most members of this superfamily is ATP hydrolysis driven unidirectional translocation of substrates (either import or export) across lipid membranes, typically in a thermodynamically unfavorable direction. However, ABC proteins also function as ion channels, regulators of ion channels, receptors, and in ribosome assembly and translation. They are structurally characterized by two highly conserved cytosolic nucleotide-binding domains (NBD) and two variable transmembrane domains (TMD) [1]–[4]. The NBDs, which are critical for ATP-binding and hydrolysis, provide the energy necessary for driving a substrate across the membrane. They are characterized by a catalytic core comprised of a Walker A motif (GXXGXGKS/T) and a Walker B motif (φφφφD; where φ represents a hydrophobic residue) separated by a conserved Q-loop and a Walker C motif. This latter component is a structurally diverse helical segment encompassing the ABC signature sequence (LSGGQ) that distinguishes ABC transporter family members from other ATP-binding proteins. Unlike the NBDs, TMDs vary in sequence, length, and helix number and are thought to provide initial substrate contact points. In eukaryotic organisms, the ABC transporter domains are organized as either full-transporters (FT) containing four domains (2 TMDs and 2 NBDs) or half-transporters (HT) comprised of only two domains (1 TMD and 1 NBD) that require homo- or heterodimerization for full functionality [2], [3].

Based on conserved domain homology and organization, the eukaryotic ABC family can be divided into eight distinct subfamilies (A-H) with most family members facilitating the movement of a diverse array of substrates (sugars, lipids, peptides, polysaccharides, metals, inorganic ions, amino acids, and xenobiotics) across membranes. The first characterized eukaryotic ABC transporter (P-glycoprotein, HsABCB1) was identified based on its multidrug efflux pump functionality in mammalian cancer cell lines [5]. Since then members of the ABCB, ABCC, and ABCG subfamilies have been reported to play roles in drug resistance and detoxification in a number of species across multiple phyla [6]–[8]. Unlike the other subfamilies, the ABCE and ABCF transporters lack TMDs and function in ribosome assembly and protein translation rather than substrate transport [9]–[13]. The ABCH subfamily, which so far has only been identified in arthropod genomes and zebrafish [8], [14], [15], has not been as extensively characterized as the other subfamilies.

Insect ABC transporters mediate diverse functions with critical roles in molting, cuticle differentiation, and egg development [16], eye pigmentation [17]–[19], uric acid uptake [20], germ cell migration [21], 20-hydroxyecdysone mediated circadian rhythmicity [22], phytochemical sequestration [23], and biogenic amine transport [24]. Insect ABC transporters also function in the clearance of xenobiotics including plant defensive compounds and numerous insecticides representing disparate chemical classes and modes of action [8], [25]. Resistance of some lepidopteran species to *Bacillus thuringiensis* (Bt) toxins has also been shown to involve novel interactions with ABC transporters independent of substrate transport [26]–[31].

Detailed genome/transcriptome wide analyses of ABC subfamilies have been conducted for a number of arthropods including water flea (*Daphnia pulex*) [32], spider mite (*Tetranychus urticae*) [33], fruitfly (*Drosophila melanogaster*) [34], olive fruit fly (*Bactrocera oleae*) [35], African malaria mosquito (*Anopheles gambiae*) [36], red flour beetle (*Tribolium castaneum*) [16], honeybee (*Apis mellifera*) [37], silkmoth (*Bombyx mori*) [37], [38], and poplar leaf beetle (*Chrysomela populi*) [39]. Among hemipterans, knowledge of ABC transporters is limited to unverified genome annotations for the pea aphid (*Acyrthosiphon pisum*) [40] and partial transcriptomic/EST analyses in whiteflies (*Bemisia tabaci*) [38], [41]–[43], bed bugs (*Cimex lectularius*) [44], and the brown planthopper (*Nilaparvata lugens*) [45].

The western tarnished plant bug (*Lygus hesperus* Knight) is a highly polyphagous hemipteran agricultural pest that primarily feeds on and injures the buds, flowers and seeds of many crops in the western United States and Canada [46]–[48]. Control strategies have traditionally relied on broad-spectrum insecticides. However, decreased efficacy has been reported for many of these compounds against field populations of *Lygus* [49]–[51]. Further compounding its pest status, our understanding of the molecular basis underlying *Lygus* resistance to insecticides is limited. To begin to address this issue, we performed a transcriptome-wide survey of ABC transporters by supplementing currently available transcriptomic data [52] with RNA-Seq analyses. We provide detailed sequence comparisons of the ABC subfamilies (ABCA-ABCH) in *L. hesperus* with those from four other arthropods and humans. In addition, the transcriptional profile of a subset of the assembled sequences was examined across developmental stages and in various sex-specific tissues. These findings will facilitate future exploration of the biological and physiological functions mediated by *L. hesperus* ABC transporters and their potential role in insecticide resistance.

## Results and Discussion

### RNA-Seq assembly and annotation

An earlier 454-based transcriptome of *L. hesperus* adults [52] contains 44 putative ABC transporter sequences. Here, we used Illumina RNA-Seq to obtain a comprehensive transcriptome that more extensively reflects ABC transporter transcription in *Lygus* adults. Furthermore, because some ABC transporters are associated with cellular stress [53]–[57], we combined the transcriptomes of *L. hesperus* exposed to cold and heat stress as well as non-stressed cohorts. Illumina HiSeq generated 144,898,116 raw 100 bp read pairs across nine libraries representing the three treatment groups. After quality filtering and *in silico* normalization, *de novo* assembly of 16,191,383 read pairs generated a raw assembly containing 132,802 isoforms across 77,246 unigenes with an N50 isoform length of 2,228 bp. Filtering this assembly by retaining only transcripts that have a predicted open reading frame reduced the assembly to 45,723 isoforms across 21,049 unigenes with an N50 isoform length of 2,989 bp.

### Identification of L. hesperus ABC transporter transcripts

The *L. hesperus* RNA-Seq database was searched using protein sequences corresponding to the full complement of ABC transporters from seven metazoans as well as the 44 putative ABC transporter sequences from the previous transcriptome [52]. We identified 65 putative *L. hesperus* ABC transporter-like (LhABC) transcripts. Based on Transdecoder predictions, significant matches with the Pfam-A database, and manual inspection of sequences spanning the first in-frame Met and stop codons, 36 of the sequences are predicted to encompass complete open reading frames. The number of LhABCs identified is comparable to that reported for other arthropods (Table 1). However, this number may under-represent (exclusion of temporally or spatially restricted transcripts not expressed) or over-represent (multiple partial transcripts corresponding to different portions or isoforms of the same gene) the actual number of LhABC transporters.

**Table 1:** ABC subfamilies in *L. hesperus* and ten other species.

phylum	Chordata	Nematoda	Arthropoda								
class					Insecta						
order			Branchiopoda	Arachnida	Diptera		Coleoptera		Hymenoptera	Lepidoptera	Hemiptera
	*H. sapiens^a^*	*C. elegans^b^*	*D. pulex^c^*	*T. urticae^d^*	*D. melanogaster^a^*	*A. gambiae^e^*	*T. castaneum^f^*	*C. pouli^g^*	*A. mellifera^h^*	*B. mori^h,i^*	*L. hesperus*
ABC subfamily											
ABCA	12	7	4	9	10	9	10	5	3	6, 9	11
ABCB	11	24	7	4	8	5	6	8	5	8, 9	6
ABCC	12	9	7	39	14	13	35	29	9	15, 15	12
ABCD	4	5	3	2	2	2	2	2	2	2, 2	2
ABCE	1	1	1	1	1	1	1	1	1	1, 1	1
ABCF	3	3	4	3	3	3	3	3	3	3, 3	3
ABCG	5	9	24	23	15	16	13	14	15	13, 12	19
ABCH	0	0	15	22	3	3	3	3	3	3, 2	11
**TOTAL**	**48**	**58**	**65**	**103**	**56**	**52**	**73**	**65**	**41**	**51, 53**	**65**

Subfamily numbers are from: ^a^ [34]; ^b^ [151]; ^c^ [32]; ^d^ [33]; ^e^ [36]; ^f^ [16]; ^g^ [39]; ^h^ [37]; ^i^ [38].

BLASTx (Table S1) and tBLASTn (Table S2) analyses revealed that the 65 putative LhABC transcripts represent all eight ABC transporter subfamilies (A-H). The most similar sequences from both BLAST analyses were putative transporters from *A. pisum* and *T. castaneum* (Figure S1). These results are consistent with previous transcriptome comparisons and not unexpected given the shared hemipteran lineage with *A. pisum* and the extensive genomic annotation in *T. castaneum*. The putative LhABC transcripts encode proteins or protein fragments ranging in size from 144 to 2237 amino acids. Although additional partial transcripts were identified in the dataset, only transcripts with coding sequences of >100 amino acids were further analyzed. Consistent with substrate translocation across the plasma membrane, 30 of the 36 full-length sequences identified are predicted to localize to the cell surface (Table 2). The six exceptions include sequences with highest similarity to ABCDs, which localize to the peroxisome, and ABCE/F subfamily members, which do not function in substrate transport. Multiple TMD prediction algorithms indicated the presence of numerous helical segments in 32 of the full-length sequences and 25 of the partial sequences (Table 2). No helices are predicted for the *L. hesperus* ABCE and ABCF transporters. ABC transporter motifs and/or NBDs are present in 62 of the 65 LhABC sequences (Table S3), and even for those lacking these domains there was significant sequence similarity (E-value<10^−12^) with genes annotated as ABC transporters (Table S1 and S2). LhABCC1A and LhABCC1B share the highest amino acid identity (96%), whereas sequence identity across the other LhABC transporters varies from 1% to 51% with highest levels of conservation observed within the respective subfamilies (Table S4).

**Table 2:** Bioinformatics analysis of putative LhABC transporters.

				Localization	Number of helical domains		
*L. hesperus* id	Illumina assembly id	Size (aa)	Full/Partial CDS	WoLF PSORT^a^	TMHMM^b^	TopPredII^c^	TopCons^d^
LhABCA1	comp10339_c0_seq1	474	partial	*nd^e^*	5	6	5
LhABCA2	comp9970_c0_seq1	663	partial	*nd*	5	5(7)^f^	6
LhABCA3	comp35546_c0_seq1	637	partial	*nd*	5	6(8)	6
LhABCA4	comp34679_c0_seq2	2237	full	PM	12	14(16)	12
LhABCA5	comp28522_c0_seq4	1326	full	PM	14	16(19)	12
LhABCA6	comp12312_c0_seq2	416	partial	*nd*	1	2	1
LhABCA7	comp33391_c0_seq1	585	partial	*nd*	5	6	5
LhABCA8	comp9781_c0_seq1	415	partial	*nd*	0	1	1
LhABCA9	comp38669_c0_seq1	275	partial	*nd*	0	0(1)	0
LhABCA10	comp39633_c0_seq1	243	partial	*nd*	0	0	0
LhABCA11	comp28530_c0_seq1	795	partial	*nd*	5	5(6)	6
LhABCB1	comp37357_c0_seq3	1191	full	PM	9	10(11)	11
LhABCB2	comp30116_c0_seq1	830	partial	*nd*	6	6	6
LhABCB3	comp31442_c0_seq2	687	partial	*nd*	5	6(7)	6
LhABCB4	comp35640_c0_seq1	834	full	PM	10	8(11)	11
LhABCB5	comp37353_c1_seq7	735	partial	*nd*	5	7	6
LhABCB6	comp21601_c0_seq1	469	partial	*nd*	6	6(7)	6
LhABCC1A	comp37257_c0_seq2	944	full	PM	9	10(12)	11
LhABCC1B	comp19288_c0_seq1	944	full	PM	7	9(12)	11
LhABCC2	comp36363_c3_seq3	589	partial	*nd*	5	5	6
LhABCC3	comp29144_c0_seq2	1407	partial	*nd*	9	11(16)	12
LhABCC4	comp26101_c0_seq1	1226	partial	*nd*	9	7(10)	12
LhABCC5	comp28277_c0_seq1	1316	full	PM	9	10(12)	12
LhABCC6	comp28206_c0_seq2	633	partial	*nd*	4	4	6
LhABCC7	comp1105_c0_seq1/comp20506_c0_seq1	601	partial	*nd*	5	5(6)	6
LhABCC8	comp36620_c0_seq1	1495	full	PM	16	14(17)	17
LhABCC9	comp33891_c0_seq2	1415	full	PM	9	10(11)	12
LhABCC10	comp14659_c0_seq1	256	partial	*nd*	0	0(1)	0
LhABCC11	comp37326_c0_seq3	856	full	PM	10	9	10
LhABCD1	comp30627_c0_seq1	676	full	cytosol	3	4(5)	5
LhABCD2	comp32676_c0_seq1	656	full	mito	5	4	5
LhABCE1	comp29836_c0_seq1	608	full	cytosol	0	1	0
LhABCF1	comp37052_c0_seq9	589	full	cyto/nucleus	0	0(1)	0
LhABCF2	comp34354_c0_seq1	629	full	nucleus	0	1	0
LhABCF3	comp24795_c0_seq2	712	full	cyto/nucleus	0	0(1)	0
LhABCG1	comp33145_c0_seq2	685	full	PM	6	6(7)	6
LhABCG2	comp28267_c0_seq1	583	partial	*nd*	6	6(8)	6
LhABCG3	comp36162_c0_seq6	622	full	PM	6	6(7)	6
LhABCG4	comp3947_c0_seq1	226	partial	*nd*	0	0(1)	0
LhABCG5	comp33057_c0_seq1	608	full	PM	5	5(8)	6
LhABCG6	comp32204_c0_seq5	617	full	PM	6	6(7)	6
LhABCG7	comp30007_c0_seq1	608	full	PM	5	4(6)	6
LhABCG8	comp26890_c0_seq1	651	partial	*nd*	7	6(9)	6
LhABCG9	comp31063_c0_seq2	479	partial	*nd*	4	4(7)	6
LhABCG10	comp32700_c0_seq1	703	full	PM	5	6(7)	6
LhABCG11	comp35069_c0_seq5	623	full	PM	6	4(8)	6
LhABCG12	comp28984_c1_seq1	654	full	PM	5	9(11)	6
LhABCG13	comp35811_c1_seq7	614	full	PM	7	7(11)	6
LhABCG14	comp26007_c1_seq1	655	partial	*nd*	6	5(6)	6
LhABCG15	comp34164_c0_seq8	601	full	PM	5	6(8)	5
LhABCG16	comp32376_c3_seq1	606	full	PM	7	6(7)	6
LhABCG17	comp32660_c1_seq2	611	full	PM	7	7(8)	6
LhABCG18	comp9896_c0_seq1	319	partial	*nd*	0	1	1
LhABCG19	comp20817_c0_seq1	227	partial	*nd*	4	4(5)	4
LhABCH1	comp36633_c0_seq2	795	full	PM	7	6(8)	6
LhABCH2	comp37335_c0_seq4	748	full	PM	5	6(7)	6
LhABCH3	comp34118_c0_seq2	683	full	PM	5	6(7)	6
LhABCH4	comp32271_c0_seq13	768	full	PM	8	8(9)	7
LhABCH5	comp31632_c0_seq1	680	full	PM	6	6(8)	6
LhABCH6	comp6701_c0_seq1	673	full	PM	5	6(7)	6
LhABCH7	comp35902_c0_seq6	685	full	PM	5	6(7)	6
LhABCH8	comp37338_c0_seq18	144	partial	*nd*	3	3	4
LhABCH9	comp22796_c0_seq1	526	partial	*nd*	0	2(3)	2
LhABCH10	comp31498_c1_seq1	693	partial	*nd*	7	7(9)	6
LhABCH11	comp27033_c0_seq3	682	full	PM	6	6(7)	6

a [155]; ^b^ [156]; ^c^ [158]; ^d^ [157]; ^e^ *nd* - not determined;

f first number indicates certain TMS (score potential >1), number in parenthesis indicates number of putative TMs.

### ABCA subfamily

ABCA transporters are among the largest known ABCs and typically exhibit an extended extracellular domain between the TMDs, a dipolar diacidic motif downstream of the NBD region, and a conserved amino terminal sequence (XLXXKN) involved in post-Golgi trafficking [58], [59]. We found 11 putative ABCA transporter sequences (Table S1 and S2) exhibiting 3–50% amino acid identity (Table S5). LhABCA4 and LhABCA5 encompass full-length coding sequences with extended extracellular domains (653 aa in LhABCA4, 189 aa in LhABCA5) and the dipolar diacidic motif. The amino terminal post-Golgi trafficking motif is only found in LhABCA4. This motif is present in 11 of the 12 HsABCAs but only 6 of 38 ABCAs from *T. urticae*, *D. melanogaster*, *B. mori*, and *T. castaneum*, suggesting that arthropods may use an alternative post-Golgi targeting mechanism.

**Figure 1:**
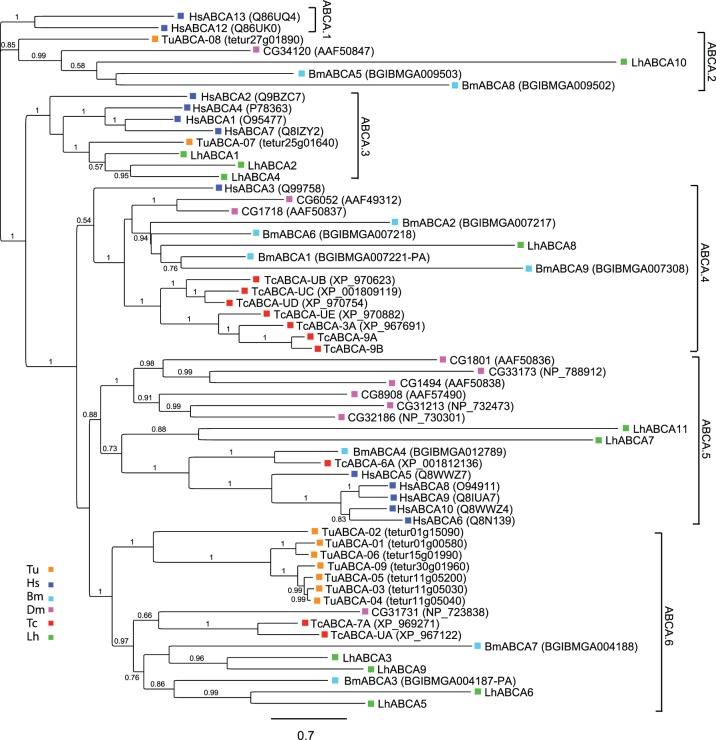
Phylogenetic analysis of ABCA transporters from *L. hesperus* and five metazoan species. Putative *L. hesperus* ABCA sequences and full-length ABCA proteins from five additional species were aligned using MUSCLE [159] and analyzed using the FastTree2 approximate likelihood method [160]. Numbers at the branch point of each node represent support values. Species abbreviations and color coding are: Bm, *Bombyx mori* (teal); Dm, *Drosophila melanogaster* (pink); Hs, *Homo sapiens* (blue); Lh, *Lygus hesperus* (green); Tc, *Tribolium castaneum* (red); and Tu, *Tetranychus urticae* (orange). Accession numbers are indicated in parentheses. The scale bar represents 0.7 amino acid substitutions per site. A full listing of the accession numbers for the five metazoan sequences is available in Table S11. LhABC transporter sequences are available in Table S12.

Alignment and phylogenetic analyses of putative LhABCAs with ABCAs from *T. urticae*, *D. melanogaster*, *B. mori*, *T. castaneum* and humans are consistent with those reported by other groups [8], [33], [37], [38] and indicate conserved subfamily clustering within six central clades that we have designated ABCA.1-ABCA.6 (Figure 1). LhABCA1, LhABCA2, and LhABCA4 aligned within clade ABCA.3, which is comprised of multiple HsABCAs that function in the transport of membrane lipids [60], [61]. LhABCA5, LhABCA6, LhABCA3, and LhABCA9 aligned with two BmABCAs to form one branch in clade ABCA.6. The first two LhABCAs are most similar to BmABCA3, while the other two share similarity with BmABCA7. Consistent with previous phylogenetic analyses [33], a second branch of clade ABCA.6 is comprised solely of *T. urticae* ABCAs. LhABCA7 and LhABCA11 form a separate branch in clade ABCA.5, which is dominated by *D. melanogaster* and human ABCAs. While no function has been assigned to the arthropod ABCAs in this clade, the HsABCAs function in lipid homeostasis [60], [61]. LhABCA10 aligned with sequences in clade ABCA.2, whereas LhABCA8 clustered with a group of BmABCAs to form a branch in clade ABCA.4. In addition to the LhABCA8 branch, the ABCA.4 clade is also characterized by gene expansion in *T. castaneum*. HsABCA3, a transporter involved in pulmonary surfactant secretion [60], [61], also sorted to ABCA.4. No arthropod sequences sorted to clade ABCA.1, which is comprised of HsABCA12, a keratinocyte lipid transporter [62], and HsABCA13, the biological function of which is not known.

### ABCB subfamily

**Figure 2:**
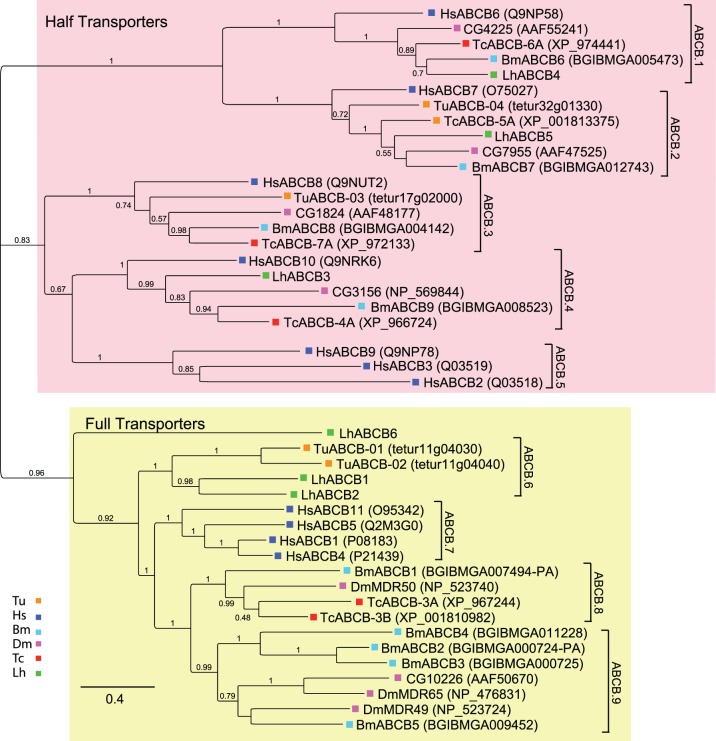
Phylogenetic analysis of ABCB transporters from *L. hesperus* and five metazoan species. Clades corresponding to full-transporters and half-transporters are shaded light yellow and light red, respectively. The scale bar represents 0.4 amino acid substitutions per site. Analyses, abbreviations, and color-coding are as in Figure 1.

ABCBs are structurally organized as either HTs characterized by two domains (1 TMD, 1 NBD) or FTs that contain four domains (2 TMDs, 2 NBDs). Mammalian ABCBs transport diverse hydrophobic substrates including bile acids, peptides, steroids, drugs, and other xenobiotics. This broad substrate range of ABCBs likely contributes to their involvement in multidrug resistance phenotypes [63]–[65] and insecticide resistance [8], [25]. Based on BLAST analyses, we identified six LhABCB transcripts, which is similar to the number reported for other arthropods (Table 1). The LhABCB1 and LhABCB4 transcripts include full-length ORFs, whereas the other LhABCB transcripts encode protein fragments ranging in size from 469 to 830 amino acids (Table 2) that encompass predicted ABC transporter-like domains (Table S3). As a group, the LhABCBs, exhibit 12–51% sequence identity (Table S6). Consistent with the phylogenetic analyses of Dermauw et al.[8], the HTs aligned into five clades (ABCB.1-ABCB.5), whereas the FTs clustered into four clades (ABCB.6-ABCB.9) largely composed of lineage specific branches (Figure 2). The branching pattern in four of the HT clades (ABCB.1-ABCB.4) is suggestive of orthologous relationships. LhABCB4, LhABCB5, and LhABCB3 aligned with proteins in clades ABCB.1, ABCB.2, and ABCB.4 respectively. The expression of HsABCB6 (clade ABCB.1) has been correlated with increased drug resistance [65], [66] and arthropod transporters in clades ABCB.1 and ABCB.2 are reported to play roles in heavy metal detoxification [67], insecticide resistance [68], cold stress tolerance [69], and pupal-adult development in *T. castaneum* [16]. Despite orthologous sequences in the other arthropods examined, no LhABCB sequences aligned with proteins comprising clade ABCB.3. The three human transporters (HsABCB2, HsABCB3, HsABCB9) that function in antigen processing [70], [71] form a human specific clade (ABCB.5).

In contrast to the HTs, the FT sequences separated into clades with species-specific branches. Two LhABCBs (LhABCB1 and LhABCB2) and two *T. urticae* ABCBs form separate branches of the ABCB.6 clade, which is consistent with diversification arising from lineage specific gene duplication events [32]. Similar to the ABCB.5 clade, ABCB.7 is human specific with no arthropod sequences aligning with the four HsABCBs. The other two FT clades (ABCB.8 and ABCB.9) comprise sequences similar to *D. melanogaster* multiple drug resistance (MDR) proteins (i.e., DmMDR49, DmMDR50, and DmMDR65) that have been identified in abiotic stress and insecticide resistance [45], [53], [72]–[78]. Surprisingly, no *L. hesperus* sequences nor any *T. urticae* sequences clustered within either of these two clades. LhABCB6, which is a partial sequence, aligned with the FTs but did not sort with any of the clades.

### ABCC subfamily

The ABCC subfamily consists of three functionally distinct classes of transporters: broad-specificity multidrug resistance-associated proteins (MRPs), sulfonylurea receptors (SUR), and the cystic fibrosis transmembrane conductance regulator (CFTR) [34], [79]–[81]. MRPs interact with a diverse array of substrates including a number of endogenous metabolites, xenobiotics, and various conjugated (glutathione, sulfate, and glucuronate) anions. Nine members of the mammalian ABCC subfamily are classified as MRPs: ABCC1–6 and ABCC10–12. In contrast to the xenobiotic transport activities of MRP-ABCCs, SURs (i.e. ABCC8 and ABCC9) function as regulators of specific potassium channels, and CFTR (i.e. ABCC7) functions as an ATP-gated chloride ion channel [34].

**Figure 3:**
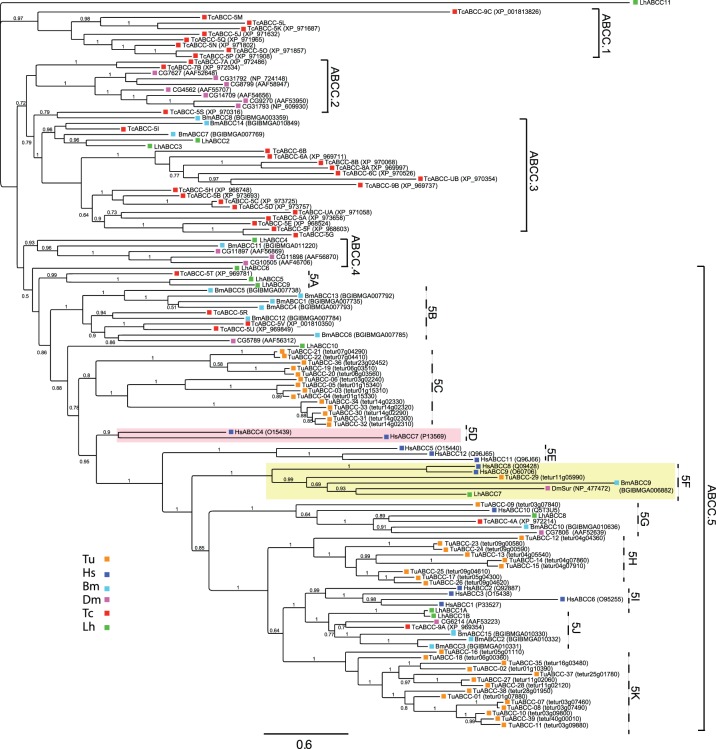
Phylogenetic analysis of ABCC transporters from *L. hesperus* and five metazoan species. The scale bar represents 0.6 amino acid substitutions per site. Clades corresponding to SUR-like sequences and CFTR are shaded light yellow and light red, respectively. Analyses, abbreviations, and color-coding are as in Figure 1.

Based on sequence similarities, we identified 12 ABCC-like transcripts in *L. hesperus*, a number comparable to that reported for other arthropods with the exception of *T. urticae*, *T. castaneum*, and *C. populi*, all of which have undergone significant expansion of the ABCC subfamily (Table 1). Sequence identity among the LhABCC transcripts ranges from 2–96% (Table S7). Half of the transcripts comprise complete coding sequences, and the rest encode protein fragments (256 to 1407 amino acids) containing ABC transporter-like domains (Table S3). Our phylogenetic analysis generated five major clades (designated ABCC.1-ABCC.5) with 11 nested clades (designated ABCC.5A-5 K) branching from ABCC.5 (Figure 3). Consistent with other studies supporting gene duplication-based expansion of the ABCC subfamily [33], [82], our analysis clustered many of the *T. castaneum* and *T. urticae* sequences into lineage specific clades/branches (*T. castaneum* – clade ABCC.1 and two nested branches in clade ABCC.3; *T. urticae* – clades ABCC.5C, ABCC.5 H, and ABCC.5 K). As before, clear relationships in sequence alignment were seen for a number of the LhABCCs.

LhABCC1A and LhABCC1B sorted to nested clade ABCC.5J. The two transcripts encode full-length sequences that are 96% identical (Table S7) with sequence variation (86% pairwise sequence identity) primarily in the first 260 amino acids (Figure S2). Sanger sequencing of multiple clones confirmed the respective sequences. The sequence variation could reflect the heterogeneity of our *L. hesperus* colony. Alternatively, they could represent splice variants similar to that reported for *D. melanogaster* ABCC CG6214 [83] and CG6214 orthologs in *A. gambiae* [36] and *Trichoplusia ni* [84]. CG6214 is most similar with the two LhABCCs in ABCC.5J and is upregulated in response to xenobiotic feeding [75], [85]. The HsABCC specific clade (ABCC.5I) that branches from the same node as ABCC.5J is comprised of classic MRPs with broad substrate specificities and diverse resistance phenotypes [80], [86].

LhABCC8 aligned to clade ABCC.5G with HsABCC10 and four other arthropod ABCCs (Figure 3). HsABCC10 transports a wide range of substrates and has been linked with multiple multidrug resistance phenotypes [87]. While the biological function of the arthropod ABCCs in this grouping is unknown, ecdysone treatment has been shown to elevate expression of the *D. melanogaster* CG7806 transporter [88]. LhABCC4 sorts to clade ABCC.4 with BmABCC11 and three *D. melanogaster* ABCCs (CG11897, CG11898, and CG10505). The expression of CG11897 is strongly induced following immune challenge with an entomopathogenic bacterium [89], whereas that of CG10505 has been linked to heavy metal homeostasis [90] and alcohol exposure [91].

LhABCC2 and LhABCC3 aligned with two BmABCCs and numerous *T. castaneum* transporters in clade ABCC.3 (Figure 3), which is a sister clade to ABCC.2. The ABCC.2 clade is characterized by a cluster of six *D. melanogaster* ABCCs, two of which (CG14709 and CG8799) are expressed and/or upregulated in response to cellular stress [92]–[94]. A third ABCC (CG4562) in that clade was recently shown to be upregulated following knockdown of a detoxifying cytochrome P450 [95], suggesting potential compensatory cross talk occurs between the two detoxification mechanisms.

ABCC2 MRP-like transporters have been linked with resistance to *Bacillus thuringiensis* (Bt) Cry1 toxins in a number of lepidopterans [26]–[31]. This resistance does not appear to be linked to ATP-dependent transport but rather to the cell surface transporter functioning as a putative Bt Cry1A toxin receptor [96]. In our phylogenetic analysis, BmABCC13 (BGIBMGA007792) and BmABCC4 (BGIBMGA007793), which represent a single gene [28], correspond to the ABCC2 transporter linked to Bt resistance. Both *B. mori* sequences align within a lineage-specific branch of clade ABCC.5B, which also included several *T. castaneum* ABCCs and a *D. melanogaster* sequence. No LhABCC sequence aligned within that clade. Although a transgenic cotton plant expressing a hemipteran-active Bt toxin has been developed [97], it remains to be determined if LhABCCs will also function as Bt toxin receptors.

Well-supported alignment of LhABCC7 with the SUR ABCC transporters in clade ABCC.5F suggests possible conservation of function. These transporters assemble with other proteins to form ATP-sensitive potassium channels that function in a number of physiological processes [34], [98]. In insects, SUR is important for glucose homeostasis [99], protection against hypoxic stress [100], and has been proposed as the putative binding site for benzoylphenylureas, a class of insecticides that inhibit chitin synthesis [16], [101]. However, recent findings suggest that in some species SUR is dispensable for benzoylphenylurea action [8], [16], [102], [103].

The third functional class of ABCC transporters is CFTR, an ABC transporter that has undergone functional divergence to exhibit ATP-gated chloride channel activity [34]. In our phylogenetic analyses, HsABCC7 (i.e., CFTR) sorted to clade ABCC.5D with HsABCC4, the closest mammalian homolog of HsABCC7 [104]. While structurally similar, HsABCC4 is functionally differentiated by a broad substrate range that includes diverse xenobiotics [80]. Consistent with other studies, we did not find any *L. hesperus* sequences orthologous with the two HsABCCs. The top BLAST hits for LhABCC11, however, are with mammalian MRP4/ABCC4 sequences (Table S1 and S2). The LhABCC11 transcript encodes an 856 aa protein (Table1) containing characteristic ABC transporter domains (Table S3), but it did not align within any of the ABCC clades. A *C. populi* ABCC transporter, CpMRP, that functions in plant-derived phenolglucoside sequestration also shares similarity with MRP4 proteins [23]. Low sequence identity (∼15%) between LhABCC11 and CpMRP, however, suggests that the two transporters may interact with different substrates.

### ABCD, ABCE, and ABCF subfamilies

**Figure 4:**
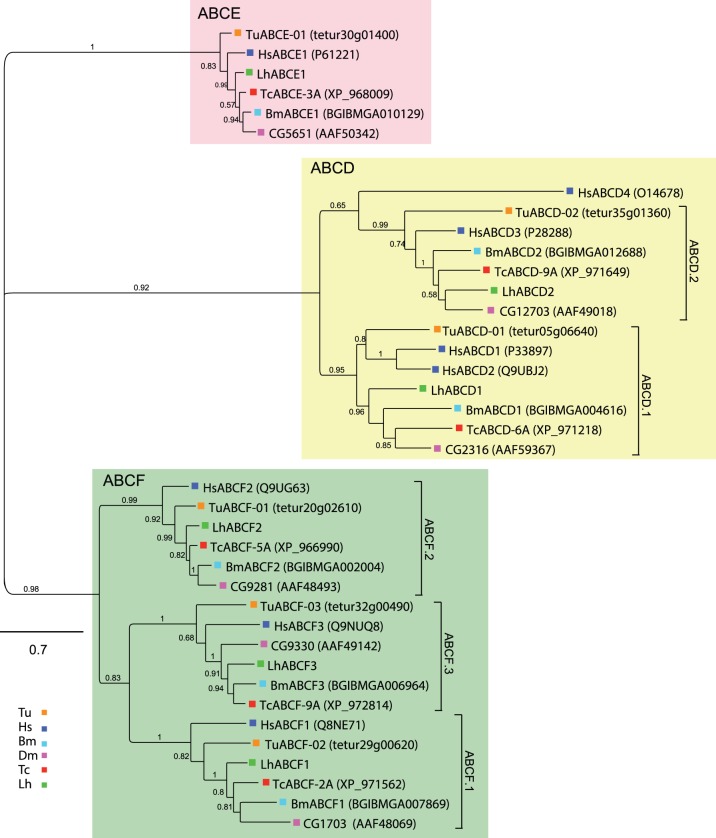
Phylogenetic analysis of ABCD, ABCE, and ABCF transporters from *L. hesperus* and five metazoan species. Clades corresponding to the three subfamilies are shaded light red (ABCE), light yellow (ABCD), and green (ABCF). The scale bar represents 0.7 amino acid substitutions per site. Analyses, abbreviations, and color-coding are as in Figure 1.

ABCD transporters function in peroxisomal import of long chain fatty acids and/or fatty acyl CoAs [105], [106]. We found that, like most other arthropods, *L. hesperus* has two ABCD transcripts (Table 1). LhABCD1 and LhABCD2 share 36% sequence identity (Table S8), which is comparable to the homology (27–35%) shared between the ABCDs in our phylogenetic analyses. Both LhABCDs have transporter domains (Table S3), as well as the two motifs, EEA-like (EEIAFYGG) and loop1 (LXXRT), that are considered essential for ABCD function [107]. LhABCD1 and LhABCD2 aligned with clades ABCD.1 and ABCD.2, respectively (Figure 4). Although little is known about arthropod ABCDs, the *D. melanogaster* ABCD transporter CG2316 (clade ABCD.1) is overexpressed in a cell line resistant to the insect growth regulator methoxyfenozide [108].

ABCE and ABCF proteins contain the characteristic NBD but lack TMDs and do not transport substrates. Instead, ABCEs function in ribosome recycling and regulation of protein translation [13], [109], whereas ABCFs are involved in translation [10], [12]. Like other arthropods [8], *L. hesperus* has a single LhABCE and three LhABCF transcripts (Table 2). LhABCE shares ∼80% sequence identity with the other ABCEs examined in our analyses, supporting a possible evolutionarily conserved role. The three LhABCFs, which share 32–35% sequence identity (Table S8), sorted into distinct but well-supported clades (Figure 4). Sequence identity within each clade ranged from 45–84%, with highest identities in the LhABCF2 clade.

### ABCG subfamily

ABCG transporters have a HT motif in which the lone NBD is localized on the amino terminal side of the TMD, in contrast to localization on the carboxyl terminal side as in other ABC transporters. In comparison with the ABCG subfamily in other metazoans, which frequently have 5–10 members [110], the arthropod lineage has undergone extensive gene expansion (Table 1). Consistent with this, we identified 19 LhABCG transcripts, including 12 full-length coding sequences (Table 2). The LhABCGs share low (10%) to moderate (58%) sequence identity (Table S9), and all but LhABCG19 have ABC transporter domains (Table S3). Despite the lack of known ABC domains, LhABCG19 shares sequence similarity (BLASTx E value<10^−10^) with other putative ABCG transporters (Table S1 and 

S2).

**Figure 5:**
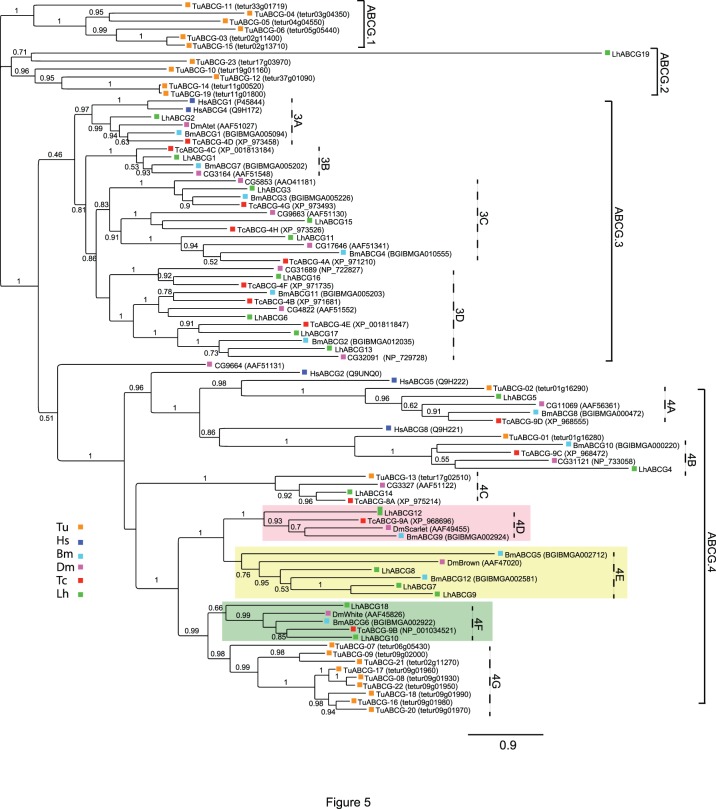
Phylogenetic analysis of ABCG transporters from *L. hesperus* and five metazoan species. Clades corresponding to transporters involved in eye pigmentation have been shaded light red (*scarlet*), light yellow (*brown*), and green (*white*). The scale bar represents 0.9 amino acid substitutions per site. Analyses, abbreviations, and color-coding are as in Figure 1.

Our phylogenetic analysis resulted in four major clades (ABCG.1-ABCG.4) with a number of minor clades branching from ABCG.3 and ABCG.4 (Figure 5). As previously reported [33], *T. urticae* has multiple lineage-specific clades (ABCG.1 and ABCG.4G) indicating expansion by multiple gene duplication events [32]. Aside from these sequences, the clustering pattern of the other ABCGs within the respective clades suggests clear orthologous relationships (Figure 5). LhABCG2 aligned to clade ABCG.3A along with *D. melanogaster* ABCG CG2969 (i.e., DmAtet), reportedly a target of the transcriptional regulator gene, *clock* [111], and two HsABCGs involved in sterol homeostasis [112], [113]. LhABCG1 sorted to clade ABCG.3B with arthropod ABCGs that have been implicated in cuticular lipid transport [16]. LhABCG3, LhABCG11, and LhABCG15 sorted to separate branches of clade ABCG.3C with LhABCG15 aligning to the same branch as the *D. melanogaster* ABCG CG9663 and LhABCG11 aligning with *D. melanogaster* ABCG CG17646. CG9663 is a putative target of *clock* [111] that has also been linked to decreased susceptibility to oxidative stress [114]. CG17646 functions in triglyceride storage [115] and ethanol sensitivity [116]. LhABCG6, LhABCG13, LhABCG16, and LhABCG17 aligned to separate branches of clade ABCG.3D with arthropod ABCGs of unknown function. LhABCG4 and LhABCG5 aligned with potential orthologs of HsABCG8 and HsABCG5 in clades ABCG.4B and ABCG.4A respectively. The obligate heterodimerization of the two HsABCGs in sterol homeostasis [112], [113] suggests similar dimerization of LhABCG5 and LhABCG4 could be important for their functionality. LhABCG14 aligned to clade ABCG.4C with arthropod ABCGs potentially involved in ecdysteroid signaling [16], [22], [117]–[119].

Six LhABCGs sorted to three clades (ABCG.4D, ABCG.4E, and ABCG.4F) characterized by *D. melanogaster* ABC transporter genes (*white, brown,* and *scarlet*) that function in the import of eye pigment precursors [17], [18]. Heterodimers of the *white* and *brown* gene products transport red-pigmented pteridine precursors, whereas heterodimers of the *white* and *scarlet* gene products are crucial for the import of brown-pigmented monochrome precursors. Consequently, *white* mutants are characterized by white eyes (complete loss of pigmentation), *brown* mutants by dark brown eyes (loss of red pigments), and *scarlet* mutants by bright red eyes (loss of brown pigments). Similar roles in pigment transport have been described for homologs of the three genes in *B. mori* [19], [20], [120], [121]. These ABCGs also function in biogenic amine transport [24], uric acid uptake [20], [121], and courtship behavior in *D. melanogaster* [122]. In our analyses, LhABCG12 clustered within the *scarlet* clade (ABCG.4D), LhABCG10 and LhABCG18 within the *white* clade (ABCG.4F), and three LhABCGs (LhABCG7, LhABCG8, and LhABCG9) that share 40–58% sequence identity (Table S9) aligned to the *brown* clade (ABCG.4E). While red eye mutants of various plant bugs, including a species sympatric to *L. hesperus* (*Lygus lineolaris*), have been reported [123]–[126], the functional importance of *scarlet* in these phenotypes is unknown. The LhABCG19 partial sequence aligned with a group from *T. urticae* in clade ABCG.2.

### ABCH subfamily

**Figure 6:**
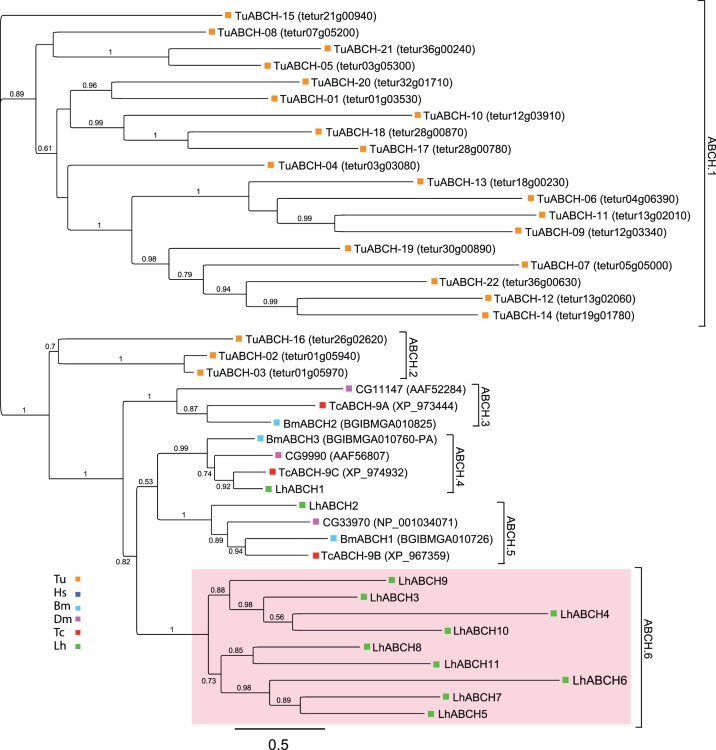
Phylogenetic analysis of ABCH transporters from *L. hesperus* and four metazoan species. The scale bar represents 0.5 amino acid substitutions per site. Analyses, abbreviations, and color-coding are as in Figure 1. Because the ABCH subfamily is largely restricted to the arthropod lineage, no representative sequences for *H. sapiens* are available and thus the analyses were performed using only arthropod sequences. *L. hesperus* ABCH sequences that have undergone gene expansion are shaded light red.

Similar to ABCGs, ABCH transporters also have the inverted NBD-TMD configuration. ABCHs though, with the exception of zebrafish [15], [127], are specific to arthropods [8], [34]. While most arthropods have 3 ABCH transporters (Table 1), lineage-specific gene duplications have resulted in 22 and 14 ABCH genes in *T. urticae* and *D. pulex* respectively [32], [33]. We found similar expansion in *L. hesperus* with 11 LhABCH transcripts, 8 of which are full-length coding sequences. Although BLASTx analyses indicate that these transcripts share sequence similarity with ABCGs (Table S1 and S2), phylogenetic analyses clustered these transcripts in the ABCH clade (Figure S3). As reported previously [33], *T. urticae* sequences are unique and do not align well with other ABCHs (Figure 6). LhABCH1 aligned with transporters in clade ABCH.4 that likely function in cuticular lipid transport [16], [128], [129]. LhABCH2 sorted to the sister ABCH.5 clade along with the *D. melanogaster* ABCH CG33970, which is upregulated in response to cold hardening [130]. The remaining LhABCH sequences formed a separate clade, implying that, like *T. urticae* and *D. pulex*, independent lineage-specific gene duplication events have contributed to the expansion of the LhABCH subfamily. While the physiological functions of the ABCH subfamily remain largely uncharacterized, differential expression of ABCHs has been reported for *T. urticae* females in diapause [119] and some ABCH transcript levels are elevated in insecticide resistant strains of *T. urticae* [33] and *P. xylostella* [131].

### Expression profile of LhABC transcripts

**Figure 7:**
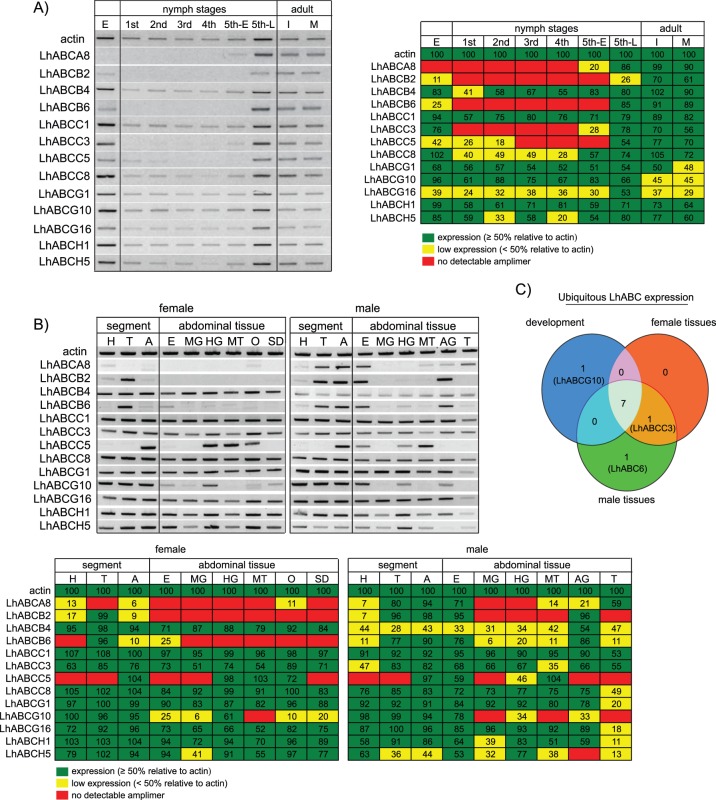
Transcriptional expression profile of 13 LhABC transcripts. A) Developmental profile. Expression profile of 13 LhABC transporters in eggs through 12-day-old adults was examined by end-point PCR using primers designed to amplify a ∼500 bp fragment of each transcript. Abbreviations: E, eggs; 1^st^, first instars; 2^nd^, second instars; 3^rd^, third instars; 4^th^, fourth instars; 5^th^-E, early stadium fifth instars; 5^th^-L, late stadium fifth instars; I, reproductively immature 1-day-old mixed sex adults; M, mature 12-day-old mixed sex adults. Products were analyzed on 1.5% agarose gels and stained with SYBR Safe. Actin was used as a positive control. Leftmost image - Representative gel image. For clarity, the negative image is shown. Rightmost image – Visual aid depicting semi-quantitative analysis of amplimers of interest compared to actin in the representative gel. Relative to the actin amplimer of each developmental stage, cells with amplimer intensity ≥50% are indicated in green, while cells <50% are indicated in yellow. Red cells indicate no detectable amplimer. Numbers inside individual cells denote the percentage of amplimer intensity compared to actin. The primer set used for LhABCC1 profiling amplifies a shared region of LhABCC1A and LhABCC1B. B) Adult tissue profile. The expression profile of the 13 LhABC transcripts was examined as above in adult body segments and various abdominal tissues prepared from 7-day-old adults. Abbreviations: H, head; T, thorax; A, abdomen; E, epidermis, MG, midgut; HG, hindgut; MT, Malpighian tubules; O, ovary; SD, seminal depository; AG, accessory glands (lateral and medial); T, testis. Top image - Representative gel image. For clarity, the negative image is shown. Lower image – Visual aid depicting semi-quantitative analysis of amplimers of interest compared to actin in the representative gel. Color shading is as above but relative to the intensity of actin amplimers within each respective tissue. C) Three-way Venn diagram comparing the transcriptional expression profile of LhABC transcripts ubiquitously expressed throughout *L. hesperus* development (blue) with those expressed in female (red) and male (green) tissues.

Many ABC transporters function in development [16], [21]. Consequently, we used end-point PCR to examine the developmental expression profile of a subset of 13 LhABCs representative of the ABCA, ABCB, ABCC, ABCG, and ABCH subfamilies. All of the transcripts were amplified from both reproductively immature and mature adults and all but LhABCA8 were expressed to some degree in eggs (Figure 7A). Five LhABCs had limited nymphal expression; LhABCA8 and LhABCC3 were detected in early and late stadium fifth instars, LhABCB2 and LhABCB6 in late stadium fifth instars, and LhABCC5 in first, second (albeit weakly) and late stadium fifth instars (Figure 7A). Orthologs of the eight LhABCs expressed throughout *L. hesperus* nymphal development have been reported to have similar expression profiles [16], suggesting potential roles in basic physiological functions.

We next examined the transcription profiles of the 13 LhABC transporters in specific body segments (head, thorax, abdomen) and abdominal tissues (epidermis/cuticle, midgut, hindgut, Malpighian tubule, ovary, seminal depository, male medial and lateral accessory glands, and testis) from 7-day-old virgins of each sex (Figure 7B). Eight of the transcripts were amplified from all tissues/segments in both sexes with seven of them also constitutively transcribed throughout development (Figure 7C). In contrast, LhABCC3, which is present in all adult tissues/segments, was only amplified from eggs, fifth instar nymphs and adults (Figure 7A), suggesting a potential role in reproductive development. The inverse was observed with LhABCG10, a *white*-like gene that is transcribed throughout development but which exhibits tissue specific transcription in adults (Figure 7B). The low abundance of LhABCG10 transcripts in Malpighian tubules differs from that reported for *white* genes in *D. melanogaster* and *B. mori* [18], [120], [132], which play key roles in the uptake and concentration of excess tryptophan [133]. The undetectable transcript levels of LhABCG10 in this tissue suggests tryptophan transport in *L. hesperus* is mediated by some other mechanism or involves a different transporter such as LhABCG18, which also sorts with *white*-like genes in clade ABCG.4F (Figure 5).

Malpighian tubules are the main excretory and osmoregulatory organs in insects and are thus crucial in clearing toxic endogenous compounds and xenobiotics [134], [135]. In support of this role, ABC transporters and detoxification enzyme levels are frequently present at relatively high levels in Malpighian tubules [75], [85], [136]–[138]. LhABCC5 expression was specific to the abdomen of both sexes where it predominantly localized to the hindgut and Malpighian tubules (Figure 7B), suggesting a potential role in xenobiotic excretion.

The expression of LhABCA8, LhABCB2, and LhABCB6 was sex-biased with higher levels of the three transcripts in male abdomen compared to female abdomen (Figure 7B). Among the male abdominal tissues, the three transporters were enriched in reproductive tissues (LhABCA8– testis; LhABCB2 and LhABCB6– accessory gland). The developmental profile of the three transcripts is likewise similar with expression limited to eggs and fifth instars (Figure 7A). This latter period coincides with the development of male reproductive organs (Figure S4), suggesting an association with sexual maturation. A number of *D. melanogaster* ABC transporters are highly expressed in male reproductive tissues [84], [132], [139] and elevated testicular expression of ABC transporters has been reported for *B. mori* [37], [38], [77]. The elevated levels of ABC transporters in reproductive tissues may be critical for protection of spermatozoa [140]. Additionally, the ABC transporters might function in the loading of accessory glands and other male secretory reproductive tissues with seminal fluid components (e.g., prostaglandins, lipids, peptides, hormones, etc.) [141], [142].

The presence of LhABC transcripts in the abdominal epidermis of both males and females (Figure 7B) could indicate potential functions in integument coloration [19], [20], [143] or in the transport of cuticular lipids to prevent water loss [16]. Alternatively, the transporters may be expressed in oenocytes, polyploid insect cells found in close association with the epidermis that have been reported to function in xenobiotic detoxification, the synthesis of cuticle components, and innate immunity [144]. Elevated levels of ABC transporter transcripts in epidermis have been reported for some insecticide-resistant bed bug populations [145], suggesting a potential role in xenobiotic transport at the cuticular layer.

## Conclusions

The genus *Lygus* encompasses more than 30 different species of polyphagous pests that attack crops worldwide. However, reports of insecticide resistance in field populations threaten the sustainability of insecticide-based management strategies. Consequently, there is growing interest in elucidating the molecular basis of resistance. While a number of studies have focused on identifying detoxification enzymes, the role of ABC transporters in insecticide clearance in hemipteran pests has been largely neglected. To address this, we used RNA-Seq to identify the ABC transporter superfamily in *L. hesperus.* Defining the functional relevance and substrate specificity of the 65 LhABC-like transcripts will be a future research priority. Initial efforts will focus on assessing the effects of insecticide exposure on expression levels of the LhABCs, in particular LhABCC5, the tissue localization of which is consistent with a role in insecticide/xenobiotic clearance. Furthermore, targeting ABC transporters by RNAi may facilitate the development of novel control strategies for *L. hesperus* and other hemipteran pests.

## Materials and Methods

### Insects

*L. hesperus* were obtained from an established laboratory colony. Stock insects were maintained at 27.5–29.0°C under 40% humidity with a L14:D10 photoperiod, and fed artificial diet packaged in Parafilm M [146]. Experimental nymphs and adults were generated from eggs deposited in oviposition packets (agarose gel packaged within Parafilm M) and maintained as described previously [147].

### RNA isolation and Illumina sequencing

To induce expression of potential stress-related genes, 10-day-old *L. hesperus* adults from a single cohort were placed individually in covered glass Petri dishes (60×15 mm) along with a section of green bean. Dishes were transferred to environmental chambers and exposed to one of three temperatures (4°C, 25°C, or 39°C) for 4 hr. Insects were stored in RNALater (Ambion, Life Technologies, Carlsbad, CA) at −80°C. Total RNA from frozen samples was isolated by the University of Arizona Genomics Center (http://uagc.arl.arizona.edu; Tucson, AZ) using an RNeasy Mini Kit (Qiagen, Valencia, CA) followed by on-column DNAse digestion according to the manufacturer’s instructions. RNA samples were eluted in 30 µL RNAse-free H_2_O. RNA quality was assessed on a Fragment Analyzer Automated CE System (Advanced Analytical Technologies, Ames, IA) and RNA was quantified using RiboGreen (Molecular Probes, Eugene, OR). Triplicate RNA libraries for each of the three temperature regimens were constructed using a TruSeq RNA Sample Preparation Kit v2 (Illumina Inc., San Diego, USA) and sequenced on an Illumina HiSeq2500 in rapid run mode. CASAVA version 2.8 was used for base calling and de-multiplexing.

### Transcriptome assembly

Raw de-multiplexed reads for each sample were assembled into single files and then trimmed and quality filtered with Trimmomatic version 0.30 [148] using the parameters LEADING:20, TRAILING:20, WINSIZE:5, WINCUTOFF:25, MINLEN:50, and ILLUMINACLIP:TruSeq3-PE.fa:2∶30∶10. Quality metrics were calculated for the unfiltered and filtered data using FASTQC version 0.10.1. After quality filtering, orphaned pairs were discarded while reads still having a read pair were used for assembly. Reads were normalized *in silico* using the “normalize_by_kmer_coverage.pl” script distributed with the Trinity transcriptome assembly pipeline (r2013_08_14) [149] and a kmer size of 25 and maximum read coverage of 40. Normalized reads were used to create a *de novo* transcriptome assembly with Trinity (Inchworm, Chrysalis, and Butterfly) using default parameters except with the jaccard_clip option used to compare paired-read consistency to reduce the creation of fused transcripts from non-strand specific data. The initial Trinity assembly was further filtered to maintain only transcripts exhibiting evidence of a coding region. Open reading frames (ORFs) were predicted using Transdecoder (r2012–08–15) with training against the 500 longest ORFs in the transcriptome. ORF transcripts were also identified based on significant matches to the Pfam-A database using a HMMER search [150]. Transcripts were only retained if they had an ORF predicted by Transdecoder with a length longer than 100 amino acids. The filtered transcriptome was annotated using InterProScan 5, and gene names assigned via BLASTp alignment to the UniProtKB/SwissProt database. The raw data was deposited in the NCBI sequence read archive under BioProject PRJNA238835, BioSamples SAMN02679940 - SAMN02679948, SRA Submission ID “PBARC: *Lygus hesperus* Heat Experiment”, SRA Study Accession SRP039607. To facilitate submission to the NCBI TSA database, transcript sequences were modified to put all coding sequence on the positive strand by reverse complementing when appropriate and the longest coding sequence for each transcript was submitted to TSA using an open source transcriptome preparation software package (http://genomeannotation.github.io/transvestigator/). The annotated assembly with putative gene name and functional annotations was submitted to NCBI under TSA submission GBHO00000000. The version described in this paper is the first version, GBHO01000000.

### Annotation and bioinformatic analysis of the L. hesperus ABC transporter superfamily

ABC transporter annotations were performed on the longest isoform for each unigene. Putative ABC transporter sequences were identified by initially performing a BLASTn search (E value ≤10^−10^) of the assembly described above with queries consisting of sequences annotated previously as ABC transporters [52]. A BLASTx search (E value ≤10^−10^) of the RNA-Seq data was also performed using the full complement of ABC transporters from *H. sapiens* [34], *C. elegans* [151], *D. pulex* [32], *T. urticae* [33], *D. melanogaster* [34], *T. castaneum* [16], and *B. mori* [37], [38]. The longest isoform of the resulting *L. hesperus* sequence hits were then re-evaluated against the NCBI nr database using BLASTx and tBLASTn (E value ≤10^−10^). The sequence list of positive hits was curated to remove duplicates.

Identification of putative ABC transporter domains was performed using ScanProsite [152], [153] and the HMMscan module on the HMMER webserver [154]. Subcellular localization prediction of LhABC transporters was performed with WoLF PSORT [155]. Transmembrane domain predictions were performed using TMHMM v2.0 [156], TOPCONS [157], and TopPred II [158].

Putative LhABC transporters were initially assigned to the respective subfamilies (A-H) based on BLAST analyses, then assignments were refined based on phylogenetic inferences. Multiple sequence alignments consisting of the putative *L. hesperus* sequences and ABCs from five metazoans (*H. sapiens*, *T. urticae*, *D. melanogaster*, *T. castaneum*, and *B. mori*) were performed using default settings for MUSCLE [159]. Phylogenetic trees were constructed utilizing FastTree2 [160] implemented in Geneious 7.0.6 using default settings augmented by the Whelan-Goldman model and optimization for Gamma20 likelihood. Parallel analyses were also performed in MEGA5 [161] with bootstrap testing of 500 replicates using both the maximum parsimony method and the maximum likelihood method based on the JTT matrix-based model [162]. Phylogenetic analyses were performed using available sequences (both complete coding sequences and partial fragments) rather than specific domains as reported for other species [16], [33]. The clustering of subfamilies and orthologous genes was compared amongst the three phylogenetic methods and with previous analyses of ABC transporters [8], [16], [33], [37], [38]. Heat identity maps for the LhABC transporters were generated using Geneious 7.1.7 (Biomatters Ltd., Auckland, New Zealand) and MUSCLE-based sequence alignments. See Table S11 for the accession numbers of the proteins used in the phylogenetic analyses, and Table S12 for LhABC transporter amino acid sequences. *T. castaneum* sequences lacking accession numbers were downloaded as FASTA protein files directly from BeetleBase (http://beetlebase.org/) based on the reported GLEAN accessions [16].

### End point PCR expression analyses

The expression profile of a subset of LhABC transporters was examined across three biological replicates throughout *L. hesperus* development and among sex-specific adult body segments/tissues. TRI Reagent Solution (Ambion) was used to isolate total RNA from pooled samples of eggs, first - fourth instars, early stadium fifth instars, late stadium fifth instars, reproductively immature adults (1-day-old) of each sex, and mature virgin adults (12-day-old) of each sex. Total RNA was also isolated from pooled 7-day-old adult virgin male and female body segments and abdominal tissues: 10×head, 3×thorax, 5×abdomen, 15×abdominal carcass, 5×midgut, 20×hindgut, 20×Malpighian tubules, 20×seminal depository, and 5 pairs each of ovaries, male medial and lateral accessory glands, and testes. First-strand cDNAs were generated using a Superscript III first-strand cDNA synthesis kit (Invitrogen) with custom-made random pentadecamers (IDT, San Diego, CA) and 500 ng of DNase I-treated total RNAs. PCR expression profiling was performed using 0.4 µL of the prepared cDNAs with Sapphire Amp Fast PCR Master Mix (Takara Bio Inc./Clontech, Madison, WI) and sequence-specific primers (Table 3) designed to amplify ∼500 bp fragments of the LhABC transcripts. Thermocycler conditions consisted of 95°C for 2 min followed by 35 cycles at 94°C for 20 s, 56°C for 20 s, and 72°C for 20 s, and finished with a 1 min incubation at 72°C. PCR products were analyzed by gel electrophoresis on 1.5% agarose gels stained with SYBR Safe (Life Tech.) and a Tris/acetate/EDTA buffer system. Representative amplimers of the expected sizes were gel excised using an EZNA Gel Extraction kit (Omega Bio-Tek Inc., Norcross, GA), sub-cloned into the pCR2.1TOPO TA cloning vector (Invitrogen), and sequenced at the Arizona State University DNA Core Lab (Tempe, AZ). In all cases, sequence variation of the cloned LhABC transporter fragments was minimal (>98% nucleotide identity) compared to the transcriptomic data, indicating that the assembled data accurately represent the sequences. The minor variations in sequence were likely attributable to the allelic heterogeneity of the *L. hesperus* laboratory colony, or to rare errors introduced during amplification.

**Table 3:** Oligonucleotide primers used in expression profiling and cloning.

gene	sequence (5′-3′)	Amplimer size
Lygus actin F	ATGTGCGACGAAGAAGTTG	555
Lygus actin R1	GTCACGGCCAGCCAAATC	
LhABCA8 8 F	AAGGCTGGTTTGCTGTGGCT	533
LhABCA8 541 R	AGGAGCTGGATCGATAGCTCG	
LhABCB2 444 F	CACCGCTCAGCAATGCAACC	491
LhABCB2 935 R	TGAGGACCTCGCCTGCGATA	
LhABCB4 1848 F	CTGGCTACGTCGTCCAGCTC	511
LhABCB4 2359 R	GTCAGCATTTCTCGCAGCGG	
LhABCB6 51 F	CCTCGCCTGGCTGAGAAGTC	481
LhABCB6 532 R	AATACAATCCGCCCGCCTCC	
LhABCC1 1240 F	GGCTGCACATACGGACTGCT	468
LhABCC1 1708 R	GCAGCACCGAAGTAGGCACT	
LhABCC3 1755 F	AGATGGCTGGGACCTTCCGT	492
LhABCC3 2247 R	TGGTTCAGAGCTGACACGGC	
LhABCC5 1143 F	GGTGGCCGAATGTCGAGTGT	523
LhABCC5 1666 R	GCGATGCTCCCCTTTCACCA	
LhABCC8 1210 F	CGTCGCAGGATTTTGCACCC	496
LhABCC8 1706 R	TGCGGTTGTCCTTGTGTTGC	
LhABCG1 220 F	TGACCATCACGCCGTGTCAG	467
LhABCG1 687 R	TCGTCTTCACGTGCTCCTGG	
LhABCG10 788 F	AAGCGTCTTGGTGGGCTCAG	517
LhABCG10 1305 R	AGGGCCCACTGACAAGGCTA	
LhABCG16 850 F	TCTGCACGATTCACCAGCCC	483
LhABCG16 1333 R	TTGCTACCGTCTTGTCCCGC	
LhABCH1 927 F	AGCTCCCCAAGTCCTGCTGA	473
LhABCH1 1400 R	CCTGTAGGGTCCCGACCGAT	
LhABCH5 934 F	ATAGGCACAGCAGTGCACCC	495
LhABCH5 1429 R	TATCCCCGGGACGTGATGCT	
LhABCC1A start F	ATGGCCGAGGATACGCTT	767
LhABCC1B start F	ATGGCAGAGGAAACACTTC	767
LhABCC1A/B 767 R	TCGCCAATGTGGCTTTCC	

## Supporting Information

Figure S1 Distribution of the most highly represented species in BLASTx and tBLASTn analyses of *L. hesperus* ABC transporter sequences. BLAST analyses were performed using the NCBI non-redundant database with an E value ≤10^−10^.
(PS) Click here for additional data file.

Figure S2 Amino acid sequence alignment of LhABCC1A and LhABCC1B. Alignment was performed using the default settings in MUSCLE [159]. Pairwise sequence identity for the full-length transporters is 96%. Pairwise sequence identity for the first 260 amino acids is 86% (223/260 aa), whereas identity over the remaining 684 amino acids is 99.6% (681/684 aa). Black shading is indicative of 100% amino acid sequence identity.
(EPS)

Figure S3 Phylogenetic analysis of ABCG and ABCH transporters from *L. hesperus* and four other species. The scale bar represents 1.0 amino acid substitutions per site. Analyses, abbreviations, and color-coding are as in Figure 1. Clades corresponding to the two subfamilies are indicated by tan shading (ABCH) or yellow shading (ABCG). As before, because the ABCH subfamily is restricted to the arthropod lineage, no representative sequences for *H. sapiens* were included in the analysis.
(EPS)

Figure S4 Length of male *L. hesperus* accessory glands and testes in fifth instar nymphs and adults. Testis length was measured from the base to the apical tip of the longest lobe. Accessory gland length was measured from the insertion at the common duct to its anterior end where the accessory gland folds over on itself. It should be noted that while primordial reproductive tissues are present in fourth instar nymphs they are smaller than that seen in early fifths and very poorly developed. Stage selection criteria were: early stadium fifth instars - small green abdomen and thin wing buds with light pigmentation; late stadium fifth instars - enlarged abdomen with yellow color and significant fatty deposits, thickened wing bugs with heavy pigmentation; adults – light body pigmentation, minimal body fat, wings not hardened, sampled within 12 h of eclosion. All specimens sampled were from the same cohort. Error bars represent standard deviation (n = 20 for each group).
(PDF)

Table S1 Top five BLASTx hits from a search against the non-redundant protein database using the 65 putative LhABC transporter sequences as a query. Analysis performed with an E value ≤10^−10^.
(XLSX)

Table S2 Top five tBLASTn hits from a search against the non-redundant database using the 65 putative LhABC transporter sequences as a query. Analysis performed with an E value ≤10^−10^.
(XLSX)

Table S3 Identification of potential protein domains in the putative LhABC transporter sequences. Analyses were performed using default settings for ScanProsite [152] and HMMScan on the HMMER webserver [154] using default settings with protein databases set to Pfam, Gene3D, and Superfamily.
(XLSX)

Table S4 MUSCLE based multiple sequence alignment heat map of the percent amino acid identities among the LhABC transporters. The matrix, which includes partial sequences, was generated from a MUSCLE alignment and indicates the percent identity across the predicted protein sequences. Cell shading is based on a sliding three color scale with lowest percent identities in red and highest percent identities in blue.
(XLSX)

Table S5 MUSCLE based multiple sequence alignment heat map of the percent amino acid identities among the LhABCA transporters. The matrix and cell shading are as described in Table S4.
(XLSX)

Table S6 MUSCLE based multiple sequence alignment heat map of the percent amino acid identities among the LhABCB transporters. The matrix and cell shading are as described in Table S4.
(XLSX)

Table S7 MUSCLE based multiple sequence alignment heat map of the percent amino acid identities among the LhABCC transporters. The matrix and cell shading are as described in Table S4.
(XLSX)

Table S8 MUSCLE based multiple sequence alignment heat map of the percent amino acid identities among the LhABCD, LhABCE, and LhABCF transporters. The matrix and cell shading are as described in Table S4.
(XLSX)

Table S9 MUSCLE based multiple sequence alignment heat map of the percent amino acid identities among the LhABCG transporters. The matrix and cell shading are as described in Table S4.
(XLSX)

Table S10 MUSCLE based multiple sequence alignment heat map of the percent amino acid identities among the LhABCH transporters. The matrix and cell shading are as described in Table S4.
(XLSX)

Table S11 Gene accession/model numbers of ABC transporter protein sequences used in phylogenetics analyses.
(XLSX)

Table S12 LhABC transporter protein sequences.
(XLS)
