# Supplementary figures and images for: Transcriptome-Based Identification of ABC Transporters in the Western Tarnished Plant Bug Lygus hesperus
Source: PLoS One. 2014 Nov 17;9(11):e113046. doi: 10.1371/journal.pone.0113046 (PMC4234516; doi:10.1371/journal.pone.0113046)

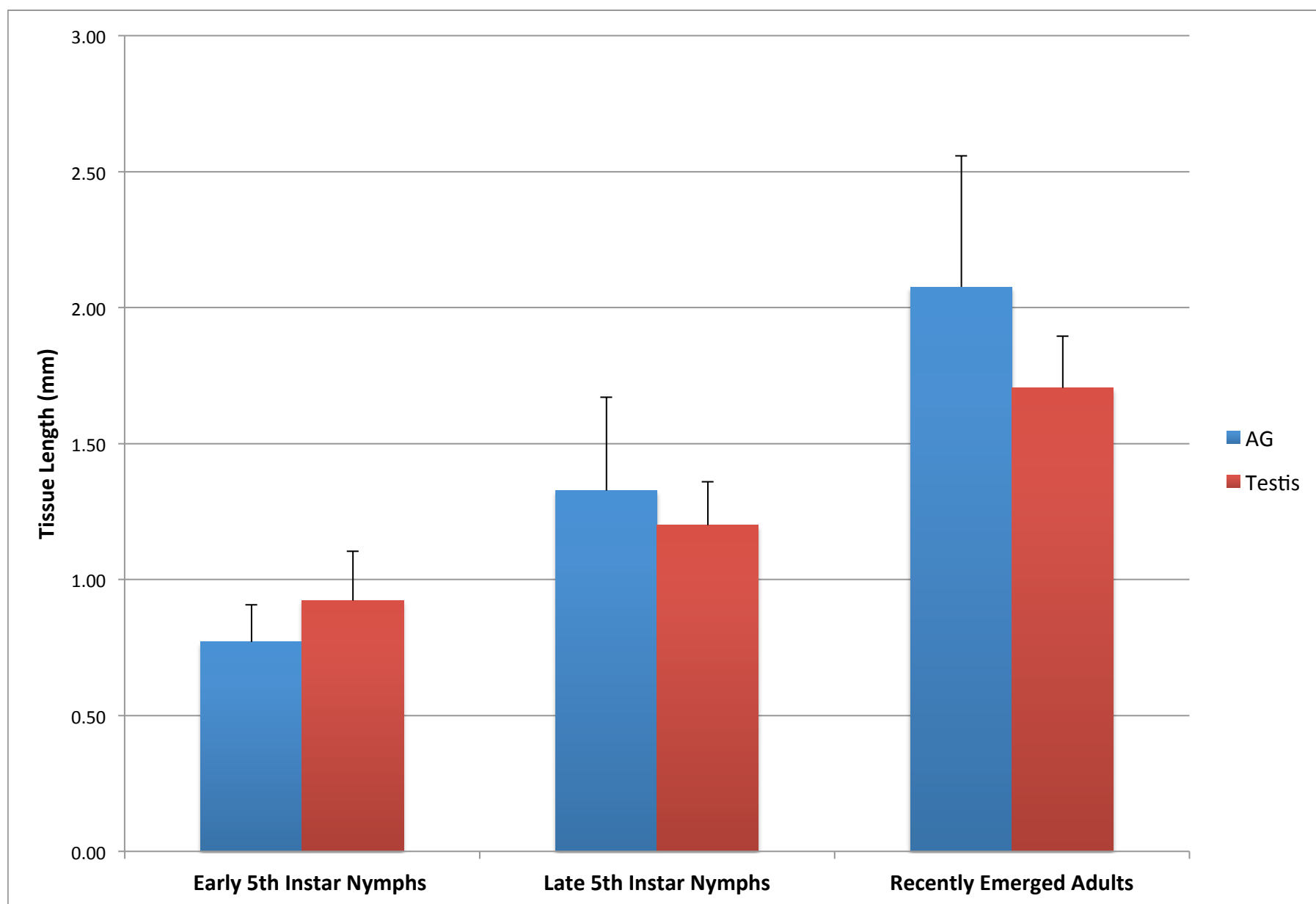

Supplement: Figure S4 — Length of male L. hesperus accessory glands and testes in fifth instar nymphs and adults. Testis length was measured from the base to the apical tip of the longest lobe. Accessory gland length was measured from the insertion at the common duct to its anterior end where the accessory gland folds over on itself. It should be noted that while primordial reproductive tissues are present in fourth instar nymphs they are smaller than that seen in early fifths and very poorly developed. Stage selection criteria were: early stadium fifth instars - small green abdomen and thin wing buds with light pigmentation; late stadium fifth instars - enlarged abdomen with yellow color and significant fatty deposits, thickened wing bugs with heavy pigmentation; adults – light body pigmentation, minimal body fat, wings not hardened, sampled within 12 h of eclosion. All specimens sampled were from the same cohort. Error bars represent standard deviation (n = 20 for each group). (PDF) [file pone.0113046.s004.pdf]
